# Higher than recommend dosage of sublingual isosorbide dinitrate for treating angina pectoris: a case report and review of the literature

**DOI:** 10.11604/pamj.2021.39.28.22180

**Published:** 2021-05-11

**Authors:** Hong-Yan Cao, Hai-di Wu, Zi-Kai Song, Ming-Long Tang, Shuo Yang, Yang Liu, Ling Qin

**Affiliations:** 1 Department of Cardiology, First Hospital of Jilin University, Changchun, China

**Keywords:** Nitrates, isosorbide dinitrate, nitric oxide, angina pectoris, case report

## Abstract

Nitrates primarily cause arterial and venous vasodilation effects, which increases coronary artery blood supply, and decreases cardiac preload and afterload by enhancing nitric oxide (NO) levels. The dosage of nitrates used for angina pectoris widely differs among individuals, and therapeutic resistance and tolerance gradually occur. Increasing doses of nitrates are needed to abolish ischemia chest pain onset in patients with angina pectoris, and to obtain satisfactory therapeutic effects. Here, we report the case of a 37-year-old male who was hospitalized six times, from September 2013 to April 2018, with recurrent angina pectoris. Although the patient was implanted with stents, he still presented with chest pain associated with physical efforts. Diagnosis with acute myocardial infarction was based on his ST-segment changes on electrocardiogram (ECG), elevated troponin-T level and coronary angiography. After the stents were implanted, his chest pain had no relief. Following three times of coronary angiography revealed that distal and small branch vessels still had stenosis, but was not required to revascularization. Due to serious headache resulted from sublingual or oral nitroglycerin; he had to take sublingual isosorbide dinitrate, from 20 mg to 150 mg each time, to obtain rapid relief from angina pectoris without doctor's consent. Followed up to April 2019, the patient has continued to take 100-150 mg sublingual isosorbide dinitrate for angina pectoris onset triggered by physical efforts, and has obtained remarkable relief within a few minutes, without blood pressure decrease and other side effects. Higher than recommend dosage of sublingual isosorbide dinitrate might establish better efficacy for angina pectoris in rarely patient.

## Introduction

Nitrates cause the dilation of both arteries and veins, and have been widely used in the treatment of coronary artery disease (CAD), angina pectoris, congestive heart failure, and hypertension for over a century. Nitrates, known as nitric oxide (NO) donors, promote NO release, and can be administered intravenously, orally, or under the tongue [1,2]. It is well known that atherosclerotic plaques in coronary arteries are often responsible for angina pectoris. Atherosclerotic plaque rupture or fissuring induces platelet adhesion and activation, leading to thrombosis within the coronary arteries and thus vessel occlusion [3]. Nitrates have shown intrinsic anti-platelet and anti-thrombotic effects, both in vitro and in vivo, which further justify their use in atherothrombotic disease [4]. Isosorbide dinitrate (ISDN) is the most commonly used nitrate, and it quickly relieves angina pectoris within a few minutes of sublingual administration. The initial sublingual dose of 5-10 mg is given for a single time. A serum peak concentration is reached in six minutes, with a half-life of 45 minutes, and for an effective duration of 10-60 minutes. In our case, for relieving angina pectoris, the dosage was increased by the patient from 5-20mg to 50mg each event, with a maximum dose of 150 mg, without the side effects of notable headaches and hypotension. We also performed a literature review and found total 7 studies regarding sublingual ISDN administration in patients with CAD (including angina pectoris and acute myocardial infarction). In reviewed literatures, sublingual ISDN exhibited good efficacy and safety, but, we have not found such high doses used like this patient (Table 1).

**Table 1:** exhibition of the reviewed studies

Number	Author/Year	Subjects (n)	Diagnosis	Dose (mg)	Approach	Results
1	Russo G /2013	24	CAD	5	Sublingual	Angina relief
2	RuDusky BM/2005	123	CAD	5	Sublingual	Reducing cardiovascular morbidity
3	Scardi S/1990	1	AMI	5	Sublingual	Angina relief
4	Figueras J/1989	43	AMI	5	Sublingual	Reducing right and left ventricular filling pressures
5	Pabón Osuna P/1985	21	AMI	10	Sublingual	No reduce infarct size
6	Durairaj SK/1980	11	AMI	5-10	Sublingual	Reduction of ischemic injury
7	Wolf R /1977	17	AMI	10	Sublingual	Reduction of ischemic size

AMI= acute myocardial infarction; CAD=coronary artery disease

## Patient and observation

**Figure 1:**
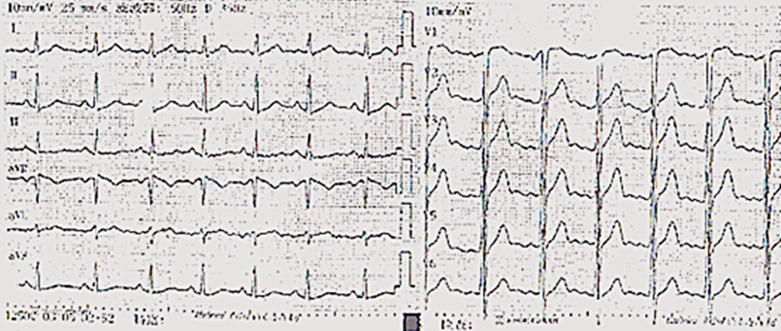
ECG was normal in September 2013

**Figure 2:**
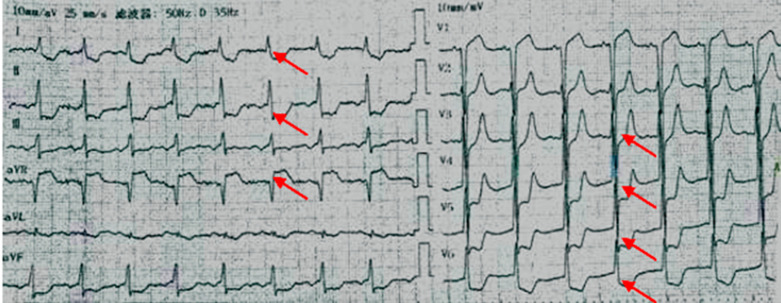
ECG showed S-T depressed more than 0.4-0.5mm on I, II, aVF, V3-V6 leads and S-T elevated more than 0.3mm on avR lead (narrows) in November 2014

**Figure 3:**
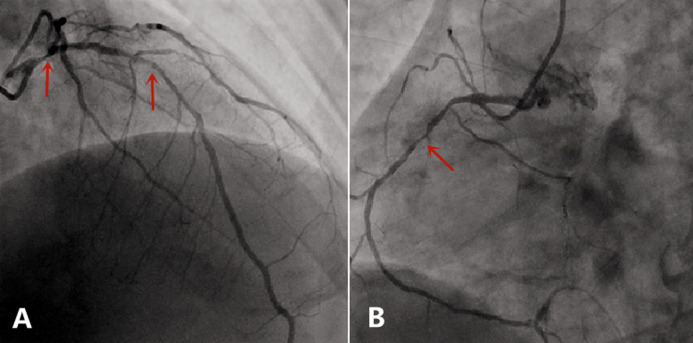
A) coronary angiography showed serious stenosis of 85-90% in LM, 70-75% in LAD in November2014 (narrow); B) coronary angiography showed 80% in proximal and distal RCA in November 2014 (narrow)

**Figure 4:**
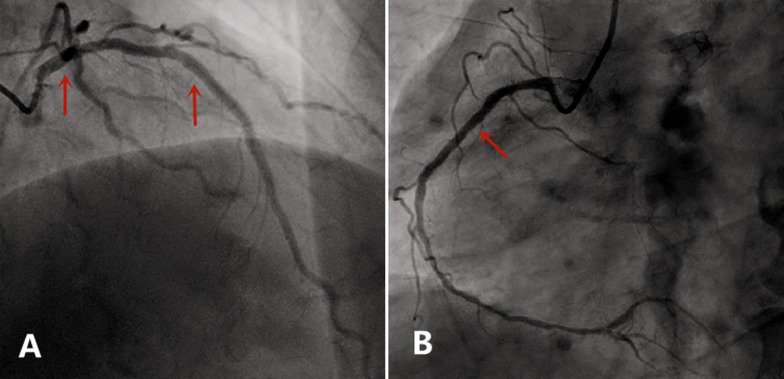
A) a drug-eluting stent was implanted in LM and two in LAD in November 2014 (narrow); B) the proximal RCA was implanted two stents in November 2014 (narrow)

**Case:** a 37-year-old male with hypertension and a history of smoking was hospitalized in September 2013 after complaints of worsening chest pain during physical exertion in the month prior to hospitalization. On admission, he was fully conscious, with a blood pressure of 160/100 mmHg and a regular heart rate of 83 bpm on auscultation. The remainder of his physical examination was normal. His electrocardiogram (ECG) (Figure 1) and his transthoracic echocardiogram were unremarkable. His test results revealed normal platelets, hemoglobin, electrolytes, liver function tests, renal function tests, N-terminal pro-brain natriuretic peptide, and cardiac troponin-T. The patient was diagnosed with primary hypertension and unstable angina. During hospitalization, the patient was administered 0.3-0.6 mg sublingual nitroglycerine, causing serious headache, and 20-30 mg ISDN under his tongue, which caused relied from chest pain within 5 minutes (Table 2). In November 2014, the patient arrived at our hospital emergency department, complaining of precordial pain that had persisted for 6 hours. At the hospital, his blood pressure was measured to be 145/90 mmHg and his heart rate was 100 bpm. Electrocardiogram showed ST-segment depression of more than 0.4-0.5 mm on I, II, aVF and V3-V6 leads, and ST-segment elevation of more than 0.3 mm on aVR lead (Figure 2). His troponin-T level was 100 times above normal (Table 2). The patient was diagnosed with ST-segment elevation myocardial infarction (STEMI) and underwent coronary angiography. The results showed serious stenosis of 85-90% in the left main coronary artery (LM), 70-75% in the proximal left anterior descending (LAD), 80-85% in the diagonal branch, and 80% in the proximal and distal right coronary artery (RCA) (Figure 3A,B). Percutaneous Coronary Intervention (PCI) was performed immediately, and one drug-eluting stent was implanted in the LM and two were implanted in the LAD (Figure 4A,B). One week after this interventional procedure, two stents were implanted in his proximal RCA (Figure 4A,B). The patient received dual anti-platelet and statin therapy regularly. After discharge, he still suffered from angina attacks, which required 50-70mg sublingual ISDN to improve the symptoms (Table 2).

**Table 2:** troponin-T, angiography, and sublingual ISDN dose

On admission	Troponin-T (< 0.010 ng/mL)	Angiography	PCI	ISDN (mg/each time)
September 2013	0.008	N	N	20-30
November 2014	1.170	Y	Stents	50-70
January 2015	0.010	Y	N	70-100
April 2016	0.005	Y	N	140
June 2017	0.003	Y	N	140
April 2018	0.003	N	N	150

N=not; PCI=percutaneous coronary intervention; ISDN=isosorbide dinitrate; Y= yes

**Figure 5:**
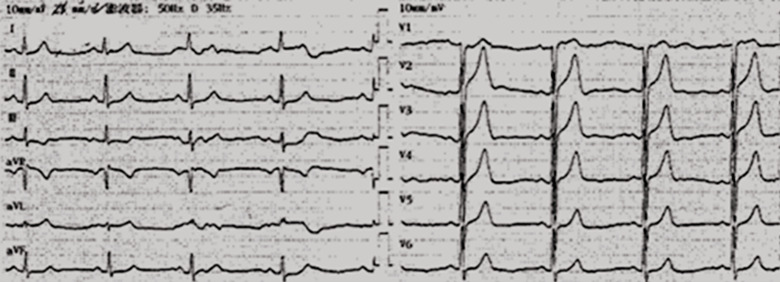
ECG was unremarkable in January 2015

**Figure 6:**
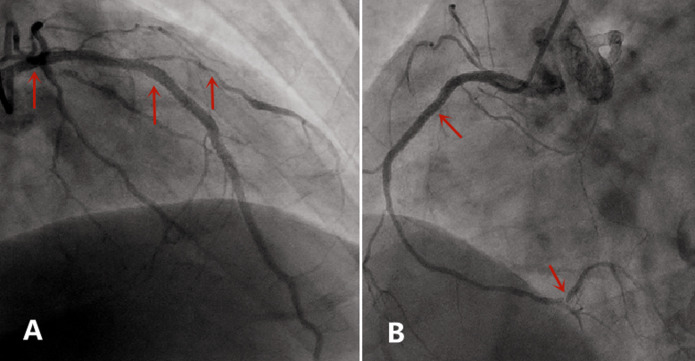
A) coronary angiography showed that no abnormality was found in the stents of LAD and diagonal branch showed still 80% stenosis in January 2015; B) coronary angiography showed that no abnormality was found in the stents of RCA and distal vessel showed 80% stenosis in January 2015

In January 2015, the patient was admitted because of aggravated precordial pain. The ECG and cardiac troponin -T level were unremarkable (Figure 5, Table 2). Reexamination of coronary angiography showed that no abnormality was present in the stents, the diagonal branch of the LAD and the distal branch of the RCA still showed 80% stenosis (Figure 6A,B) Because the blood vessels were small, invasive intervention was not performed. A sublingual ISDN dosage of 70-100 mg was effective in relieving onset of chest pain out-hospital (Table 2). The patient was again admitted to our hospital in April 2016 and June 2017 with complaints of chest pain, and coronary angiography showed no change in condition compared with that seen in January 2015 (Figure 6A,B). In June 2017, the dosage of sublingual ISDN was increased up to 140 mg in order to relieve angina onset. In April 2018, the patient was hospitalized for chest pain again, and his ECG had no changes than before and troponin -T level showed no abnormalities (Table 2). During follow-up to April 2019, the patient also used 50-150 mg sublingual ISDN, which was effective in relieving angina onset within a few minutes without blood pressure decrease. Our case report was waived from the First Hospital of Jilin University Ethical Board, based upon their policy to review all intervention and observational study except for a case report. The patient provided informed consent for the publication of his clinical data. The presented data are anonymized and risk of identification is minimal.

**Literature review:** we searched terms 'sublingual isosorbide dinitrate; angina pectoris; coronary artery disease; myocardial infarction' in PubMed and Web of Science databases to identify relevant English-language studies. Reports among this relevant literature were presently reviewed to understanding the dosage of sublingual ISDN in patients for relieving angina pectoris attack and efficacy. Seven studies were found which were a case report and 6 clinical studies (Table 1). Two hundred and forty (240) patients were enrolled in 7 studies. Patients in 2 studies were diagnosed CAD [5,6], and in 5 studies were diagnosed acute myocardial infarction [7-11]. All patients received sublingual ISDN 5-10mg each time, fewer patients presented mild headache. Two study results revealed good effects in relieve angina pectoris onset [5,6]. The effectiveness of reducing right and left ventricular filling pressures [8] and reduction of ischemic injury [10], ischemic size [11] and mortality [6] were reported respectively. A study enrolled 21 patients showed that sublingual ISDN did not reduce infarct size [9].

## Discussion

Coronary artery disease (CAD) is the leading cause of mortality globally [12]. Organic nitrates, the second oldest cardiovascular drug class after digitalis alkaloids, have been commonly used for more than a century. Recently, 2019 ESC Guidelines for the diagnosis and management of chronic coronary syndromes still recommended nitrates used for anti-angina therapy [13]. Despite the advent of new cardiovascular therapeutic agents in the modern era, organic nitrates remain one of the major classes of medications for managing CAD, especially for acute cases, and consistent recommendations and guidelines have been set for their use. The beneficial pharmacological effects of nitrates have been largely attributed to their vasodilation and hemodynamic effects. Nitrates have recently been shown to have anti-platelet effects, which has further justified their use in the treatment and prevention of cardiovascular disease [13]. In normal blood vessels, the intact endothelium releases NO, which activates guanylyl cyclase. This increases the levels of cyclic guanosine monophosphate in smooth muscle cells and causes vasodilation, resulting in the prevention of coronary vasospasm and platelet adherence and activation [14]. This, in turn, reduces vessel wall stress and myocardial oxygen demand, and improves cardiac parameters and exercise tolerance [15]. In diseased vessels, the release of NO from endothelial cells is impaired and the aggregation of platelets tips the balance toward thrombus formation in these vessels [16]. A recent study found that antecedent nitrate therapy may offer protection from acute ischemic events via preconditioning [17]. Isosorbide dinitrate, like nitroglycerin, plays a unique anti-angina role by mediating NO release, and it can be administered intravenously, under the tongue, or orally. Sublingual administration acts faster than oral administration. It is completely absorbed under the tongue, free from the influence of food, metabolized through the liver, and eventually excreted by the kidneys. The main side effects are hypotension and headache.

We searched in PubMed and Web of Science databases relevant studies. In 7 studies involved 240 patients, all patients who were prescribed sublingual ISDN were 5-10 mg each time. 147 patients with CAD in two studies were observed better efficacy for relieving angina and improving S-T depression in ECG [5,6]. In remaining 97 patients, 76 patients diagnosed acute myocardial infarction manifested reduction of right and left ventricular filling pressures, improvement of ischemic injury and ischemic size and decrease mortality [6-8,10,11]. Only a study enrolled 21 patients showed that sublingual ISDN did not reduce infarct size [9], and fewer patients presented mild headache. Nitroglycerin is a short-acting nitrate for acute effort angina. Sublingual and spray nitroglycerin formulations provide immediate relief of effort angina. Nitroglycerin can be administered for prophylaxis before physical activities known to provoke angina. Isosorbide dinitrate (5mg sublingually) has a slightly slower onset of action than nitroglycerin due to hepatic conversion to mononitrate [13]. Therefore, in clinical practice, nitroglycerin was firstly selected anti-angina pectoris [18]. Our patient experienced typical ischemic chest pain, showed progression from the ischemia phase to acute myocardial infarction phase, and subsequently recovered back to ischemia phase, during a span of over five years. Because of his severity headache for nitroglycerin, he received over-dosages ISDN for solving ischemia chest pain without physician´s suggestion and did not present any side effects.

## Conclusion

In both literature review and our clinical practice, we present the first report of the anti-angina pectoris effects of extremely overdoses of sublingual ISDN. So far, it is unclear why the patient can tolerate such dose and achieve good relief, at same time, there were no side effects. We consider that intolerance dosage might vary greatly among individuals, rarely individual might resist to the drug during long-term use. The resistance might improve by increasing doses. Blood concentration and pharmacokinetics should be monitored in such rare case. Such a dose cannot be recommended in clinical procedures.
